# Noncanonical Constitutive Androstane Receptor Signaling in Gene Regulation

**DOI:** 10.3390/ijms21186735

**Published:** 2020-09-14

**Authors:** Yuliya A. Pustylnyak, Lyudmila F. Gulyaeva, Vladimir O. Pustylnyak

**Affiliations:** 1 Zelman Institute for the Medicine and Psychology, Novosibirsk State University, Pirogova street, 1, 630090 Novosibirsk, Russia; yupustylnyak@mail.ru (Y.A.P.); gulyaeva@niimbb.ru (L.F.G.); 2 Federal Research Center of Fundamental and Translational Medicine, Timakova street, 2/12, 630117 Novosibirsk, Russia

**Keywords:** CAR, noncanonical signaling, gene regulation, energy homeostasis, cell proliferation

## Abstract

The constitutive androstane receptor (CAR, NR1I3) is extremely important for the regulation of many physiological processes, especially xenobiotic (drug) metabolism and transporters. CAR differs from steroid hormone receptors in that it can be activated using structurally unrelated chemicals, both through direct ligand-binding and ligand-independent (indirect) mechanisms. By binding to specific responsive elements on DNA, CAR increases the expression of its target genes encoding drug-metabolizing enzymes and transporters. Therefore, CAR is mainly characterized as a ligand-dependent or ligand-independent transcription factor, and the induction of gene expression is considered the canonical mode of CAR action. Consistent with its central role in xenobiotic metabolism, CAR signaling includes a collection of mechanisms that are employed alongside the core transcriptional machinery of the receptor. These so-called noncanonical CAR pathways allow the receptor to coordinate the regulation of many aspects of cell biology. In this mini-review, we review noncanonical CAR signaling, paying special attention to the role of CAR in energy homeostasis and cell proliferation.

## 1. Introduction

The constitutive androstane receptor (CAR, NR1I3) is a member of the nuclear receptor superfamily (NR), which includes steroid, retinoid, and thyroid hormone receptors [1]. Members of this superfamily play key roles in almost all aspects of development and physiology, as they function as ligand-activated transcription factors. CAR belongs to the vitamin D receptor subfamily of the nuclear receptor superfamily [1]. CAR is mostly expressed in the liver [2].

CAR, similar to most members of the superfamily, has a classic domain structure that includes a DNA-binding domain (DBD) with two zinc fingers and a conserved ligand-binding domain (LBD). DBD is involved in the interaction of the receptor with short stretches of DNA, termed response elements, in the regulatory regions of target genes. LBD serves as a ligand docking site and also contains dimerization motifs and transcription activation domains, such as the region of activation function 2 (AF-2) [3]. The AF-2 region of CAR contains a very stable helix H11 in its structure. Therefore, AF-2 is constantly fixed in the active conformation [4]. Interaction with ligands further enhances receptor activity [5].

CAR is activated by various chemical compounds that are related to drugs, pesticides, food flavonoids, polyphenols, etc. [6,7]. The half maximal effective concentrations (EC50s) for ligand binding to CAR range from the nanomolar to micromolar range, suggesting that even low-affinity binding of ligands to CAR can cause strong cellular signals. The ability of some ligands to bind to the receptor remains a matter of discussion, since only two compounds—1,4-bis-[2-(3,5-dichloropyridyloxy)] benzene (TCPOBOP) and 6-(4-chlorophenyl) imidazo-[2,1-b][1,3] thiazole-5-carbaldehyde-O- (3,4-dichlorobenzyl)oxime (CITCO)—display a direct interaction with the CAR ligand-binding pocket. The affinity of these compounds for CAR varies significantly between species. TCPOBOP activates CAR in mice, but not in humans, while CITCO has the opposite effect. In addition, CAR can be activated by chemical compounds indirectly, through signal transduction pathways. A classic example of an indirect CAR activator is phenobarbital (PB), which triggers a signaling cascade in hepatocytes that leads to the CAR active state [8].

CAR was originally characterized as a xenosensor that induces the expression of the *CYP2B* gene (cytochrome P450 of the 2B subfamily), which is involved in the biotransformation of a wide range of xenobiotics, including drugs [9,10]. It was later demonstrated that CAR regulates many genes encoding key enzymes of drug and xenobiotic metabolism, including phase I (*CYP2B*, *CYP3A*, etc.), phase II (*SULT*, *UGT*, and *GST*), and transporter genes [11]. Drug-metabolizing genes are regulated by CAR according to the canonical mechanism: CAR binds to the phenobarbital (PB)-responsive enhancer module (PBREM) in the promoter of the target gene, activating its expression [9,10].

It was previously shown that the activation of CAR has a pleiotropic effect on physiological and pathological processes: activation of CAR alters glucose homeostasis and lipid metabolism and also leads to cell cycle disturbances and the inhibition of apoptosis. Using RNA-seq technology, it was found that CAR activation causes a change in the expression of more than 2000 genes [12]. CAR activation can lead to both the induction and inhibition of gene expression. For example, the activation of CAR leads to a decrease in the expression of gluconeogenesis genes in the liver [13,14]. In addition, CAR activation significantly enhances hepatocyte proliferation, followed by liver hyperplasia [15]. Often, the regulation of processes, other than drug metabolism and elimination, is carried out by noncanonical CAR signaling. This mini-review contains the recent progress in our understanding of noncanonical CAR signaling and how it coordinates the regulation of several aspects of the biochemistry of cells.

## 2. Activation of CAR Nuclear Translocation

Compared to other nuclear receptors, CAR has a unique activation mechanism, which includes both nuclear translocation and nuclear activation [16,17]. In addition, another feature of the activation of CAR is the fact that a number of compounds that activate the transcriptional activity of the receptor are not its ligands [18]. Therefore, the direct interaction of CAR with these chemical compounds is not necessary for its activation [19]. In the inactive state, CAR is located in the cell cytoplasm in a complex with several proteins: heat shock protein 90 (HSP90), cytoplasmic CAR retention protein (CCRP), and the membrane-associated subunit of protein phosphatase 1β (PPP1R16A) [20,21,22,23]. In addition, this complex is stabilized in an inactive state by the HSP70 chaperone protein [24]. When CAR interacts with a ligand in the cytoplasm, the chaperone proteins HSP90 and CCRP, which maintain the inactive state of the receptor, dissociate and CAR translocates to the nucleus.

Phenobarbital (PB) is a classic example of an indirect CAR activator, which exerts its activating effect through signal transduction pathways. It has been demonstrated that the activation of CAR using PB is mainly associated with its nuclear translocation. Moreover, nuclear translocation of the receptor is reduced by a protein phosphatase 2A inhibitor [10]. This suggests that the nuclear translocation of CAR, caused by an indirect activator, is associated with the signaling pathway in which dephosphorylation of the receptor protein occurs. It was further shown that activation of the protein kinase ERK1/2 leads to the inhibition of CAR nuclear translocation and inhibition of the transcription of CAR target genes. Inhibition of the ERK1/2 signaling pathway enhanced the induction of CAR target genes [25]. To activate hCAR nuclear translocation under the action of PB, phosphorylation of the receptor in Thr38 (Thr48 in mCAR) is necessary [26]. In 2013, it was convincingly demonstrated that PB exerts its activation effect through a pathway involving the epidermal growth factor receptor (EGFR) [8]. It has been shown that PB is able to bind to EGFR and block the activation of its signaling pathway. This leads to the activation of protein phosphatase 2A (PP2A), which dephosphorylates CAR to the Thr38 position and triggers its nuclear translocation.

## 3. Canonical CAR Pathway

Through the canonical signaling pathway, CARs increase the expression of target genes encoding drug-metabolizing enzymes and transporters. The marker CAR target gene is *CYP2B*, whose expression upon receptor activation increases to a much greater extent than that of other genes. In 1995, it was demonstrated that the response to PB is associated with a nucleotide sequence located in the region of -2318/-2155 bp in the *CYP2B2* gene promoter in rat hepatocyte cultures [27]. Later, in experiments on cultures of mouse hepatocytes conducted in the laboratory of M. Negishi, a 51 bp sequence was identified in the regulatory region of the *Cyp2b10* gene. The introduction of mutations in this region led to a loss of sensitivity to PB. The sequence was termed PBREM [28]. The sequence has also been found in other species, such as rats and humans [29]. PBREM consists of two nuclear receptor binding sites (NR1 and NR2) and a nuclear factor 1 binding site (NF1) [9]. NR1 and NR2 contain incomplete direct repeats separated by four base pairs (DR4). The sequence of NR1 (5′-TGTACTTTCCTGACCT-3′) in the promoter region of *CYP2B* genes in different species is the most conserved [30]. 

In a further series of experiments, using affinity chromatography with an NR1 oligonucleotide as a ligand, CAR was identified as a key induction factor under the action of phenobarbital [9]. It was shown that in fractions obtained from extracts of the liver nuclei of mice treated with PB, CAR accumulates together with retinoid X receptor (RXR, NR2B). In the nucleus, CAR heterodimerizes with RXR and recruits coactivators, which leads to interactions with PBREM in the regulatory regions of target gene promoters [19]. In 2003, an additional CAR-specific regulatory element was identified in the *CYP2B6* gene promoter. This regulatory sequence was termed the xenobiotic responsive enhancer module (XREM), and is located in the region of −8500 bp in the promoter of *CYP2B6* gene [31]. XREM contains a cluster of CAR binding sites, which, by analogy with the NR1 and NR2 of PBREM, were termed NR3–NR8. Both PBREM and XREM are required for maximum activation of the *CYP2B6* gene in human hepatocytes.

The main function of coactivators is to alter the chromatin structure, which facilitates the interaction of the general transcription apparatus for induction of transcription of CAR target genes. The key role among coactivators belongs to Steroid Receptor Co-activator-1 (SRC-1), Glucocorticoid Receptor Protein-1 (GRIP-1), and Proliferator-activated receptor Gamma Coactivator-1 alpha (PGC-1α) [32,33,34,35]. In vitro, it was shown that the transcription factor Sp1 can act as a coactivator of CAR in the upregulation of *CYP2B* gene expression [31].

According to this canonical mechanism, the activation of CAR leads to an increase in the expression of genes involved in the metabolism and elimination of a wide range of xenobiotics. These genes include members of the cytochrome P450 superfamily, glutathione-S-transferases (*GSTs*), sulfotransferases (*SULTs*), UDP-glucuronyltransferases (*UGTs*), and transporters [11,36].

## 4. Noncanonical CAR Signaling in Gluconeogenic Gene Regulation

Recent studies have demonstrated new CAR functions in various cell processes, for example, glucose metabolism and the regulation of hepatocyte proliferation. Many CAR functions in the liver, other than the regulation of drug-metabolizing genes, are mediated by noncanonical signaling and result in the downregulation of gene expression. The most studied is the CAR-mediated downregulation of gluconeogenesis genes. It is well-known that the use of PB in patients with diabetes leads to a decrease in blood glucose [37]. It was demonstrated that PB downregulates the expression of genes that encode rate-limiting enzymes of hepatic gluconeogenesis: phosphoenolpyruvate carboxykinase (*PEPCK*) and glucose-6-phosphatase (*G6Pase*) in a CAR-dependent manner [38]. In addition, CAR activation reduces hyperglycemia and increases insulin sensitivity in mice with metabolic disorders [39,40,41]. Given the important effects of CAR activation on metabolic processes, CAR can be considered an attractive therapeutic molecular target for the treatment of metabolic disorders.

**Figure 1:**
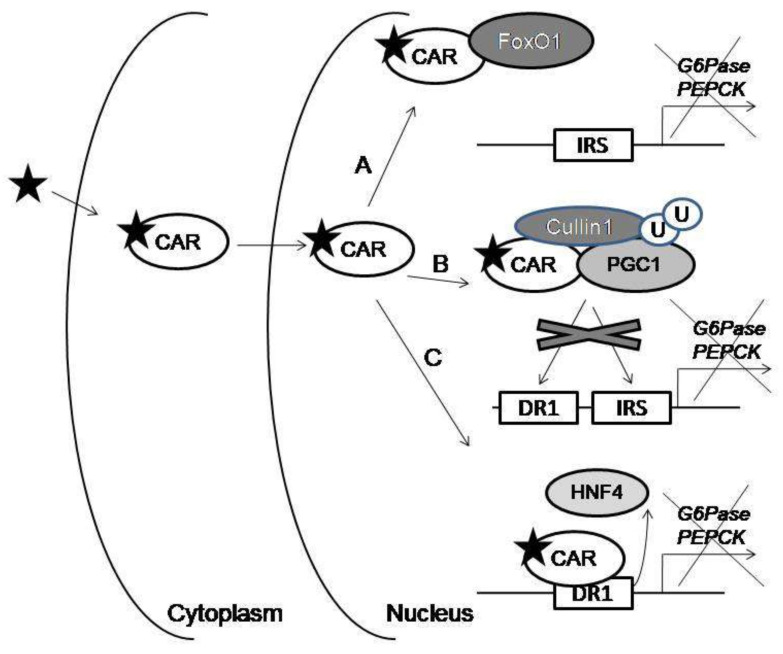
Noncanonical mechanisms of the constitutive androstane receptor (CAR)-mediated suppression of gene expression. When CAR interacts with a ligand in the cytoplasm, CAR translocates to the nucleus. (**A**) Activated CAR binds to forkhead box O1 (FoxO1), thereby blocking its interaction with the insulin-responsible sequence (IRS) in the gene promoters. (**B**) Activated CAR serves as an adapter protein for the recruitment of Proliferator-activated receptor Gamma Coactivator-1 alpha (PGC1α) into the complex with E3 ligase Cullin1, where PGC1α undergoes ubiquitination and subsequently, proteasome degradation. (**C**) Activated CAR competes with hepatic nuclear factor-4α (HNF4α) for binding to DR1.

In recent years, several studies have been carried out that have described in detail three possible mechanisms underlying the regulation of gluconeogenesis by CAR activators. In these experiments, it was demonstrated that CAR is able to regulate the expression of gluconeogenesis genes via noncanonical pathways involving protein–protein interactions. Insulin has an inhibitory effect on the transcription of the *G6Pase* and *PEPCK* genes in which rate-limiting enzymes are encoded. The expression of these genes is regulated by the transcription factor forkhead box O1 (FoxO1), which binds to the insulin-responsible sequence (IRS). Insulin activates protein kinase B (Akt), which phosphorylates FoxO1. Such phosphorylation leads to the transfer of FoxO1 from the nucleus to the cytoplasm and its proteosomal degradation [42]. In vitro and in vivo experiments showed that activated CAR binds to FoxO1, thereby blocking its interaction with IRS in the regulatory regions of the *PEPCK* and *G6Pase* genes [43,44] (Figure 1A).

PGC1a is also a major regulator of gluconeogenesis [45]. FoxO1 and PGC1a cooperate to induce gluconeogenesis by activating *PEPCK* and *G6Pase* gene expression [46]. It was previously shown that CAR suppresses the expression of gluconeogenic genes through post-translational regulation, subcellular localization, and degradation of the PGC1α coactivator (Figure 1B). Activated CAR translocates to the nucleus and serves as an adapter protein for the recruitment of PGC1α into the complex with E3 ligase Cullin1 [47]. After that, PGC1α undergoes ubiquitination and, subsequently, proteasome degradation. It has been suggested that such negative regulation of PGC1α by CAR could be a cellular adaptive mechanism for adapting to energy-limited conditions. Therefore, the protein–protein interactions described above underlie the suppression of the expression of key genes involved in gluconeogenesis in response to the action of CAR activators.

In addition to FoxO1, other transcription factors, such as hepatic nuclear factor-4α (HNF4α), regulate *G6Pase* and *PEPCK* gene expression [48]. Both genes contain functional HNF4α-binding sites (DR1 motif) in their regulatory regions. The third noncanonical mechanism of the regulation of gluconeogenic genes mediated by CAR is associated with the transcription factor HNF4α. CAR first competes with HNF4α for binding to DR1. The binding of activated CAR to DR1 leads to the repression of transcription of the *G6Pase* and *PEPCK* genes [49,50,51] (Figure 1C). Secondly, CAR competes with HNF-4α for a limited pool of common coactivators, including PGC-1α, which dissociate from promoters of the HNF-4α target genes [49].

Recently, the existence of gender differences in CAR-mediated regulation of energy homeostasis has been demonstrated [52]. Understanding the role of sex hormones in the CAR-mediated mechanism underlying sexual dimorphism in glucose homeostasis may facilitate the development of sex-specific therapy for metabolic diseases.

## 5. Noncanonical CAR Signaling in Hepatocyte Proliferation

It has been known for a long time that the activation of CAR is accompanied by a strong proliferative effect in hepatocytes [53,54,55]. This fact gave grounds to consider CAR as a therapeutic target for partial liver resection [56]. Moreover, in 2016 it was demonstrated that in CAR-/- mice, liver failure occurs even after standard hepatectomy (2/3 of the liver). Pharmacological activation of CAR in wild-type mice can improve liver regeneration and inhibit the development of liver failure during extreme resections (more than 85% of the organ), and this effect is mediated by a decrease in the level of p21 protein [57]. Several studies have demonstrated a possible mechanism for CAR-mediated regulation of p21 levels. A key role in the activation of liver hyperplasia is played by promitogenic protein cMyc signaling, which promotes hepatocyte proliferation [58]. However, the question of how CAR regulates the level of cMyc in response to TCPOBOP treatment remained has unanswered for a long time, since a functional CAR binding site has not yet been identified in the *cMyc* gene promoter [58]. It has recently been demonstrated that PB is able to reduce miR-122 in mouse livers [59]. Moreover, in the same work, it was shown that PB inhibits the transactivation of the *pri-mir-122* promoter. This suggests that the suppression of miR-122 upon exposure to PB occurs at the level of transcription. MiR-122 is a liver-specific miRNA that accounts for about 70% of the miRNA population in this organ [60] and plays a significant role in many physiological processes in the liver [61]. MiR-122 regulates cMyc through regulation of the level of transcription factor E2f1 [62]. Transcription of *pri-miR-122*, the precursor of miR-122, is regulated by hepatic transcription factors, including HNF4α [63,64]. HNF4α increases the miR-122 level by directly binding to the regulatory region of *pri-miR-122* [64]. As was the case with the regulation of gluconeogenic genes, it was demonstrated that CAR decreases the level of miR-122, which is the molecular target of the transcription factor HNF4α, competing with HNF4α for binding to the DR1 motif in the *pri-miR-122* promoter [65]. The decrease in miR-122 caused by CAR activation is accompanied by an increase in E2f1, as well as its accumulation on the *cMyc* promoter. CAR activation decreases miR-122 through the suppression of HNF4α transcriptional activity on the *pri-miR-122* promoter and indirectly regulates cMyc. Moreover, a CAR-mediated decrease in miR-122 could produce activation of the Akt through initiation of the cMyc-FoxM1-Nedd4-1-PTEN pathway [66] and subsequent Akt-Foxo1-mediated decrease of p21 [67]. Therefore, the noncanonical CAR signaling in gene regulation, based on competition with HNF4a for the DR1, appears to be involved not only in the regulation of gluconeogenesis but also in the regulation of hepatocyte proliferation.

Understanding the molecular mechanisms of CAR-mediated induction of hepatocyte proliferation is very important, since the loss of the ability of hepatocytes to maintain a balance between growth stimulating and inhibitory signals may be a trigger for tumor promotion. In 2004, the role of CAR in hepatocarcinogenesis was first demonstrated using wild-type and CAR -/- mice. It has been demonstrated that chronic administration of PB induces liver tumors in wild-type mice [68]. Recently, it was shown that the hepatocarcinogenic effect of CAR activators appears in combination with β-catenin [69,70]. There is evidence that CAR-mediated activation of the Akt pathway causes redistribution of β-catenin from the cytoplasm to the nucleus in dividing hepatocytes [71]. CAR activates yes-associated protein (YAP) signaling, which may also be involved in CAR-mediated liver carcinogenesis [72,73]. The question of how liver tumors are promoted upon activation of CAR remains open. 

## 6. Conclusions

CAR was originally characterized as a xenosensor that, when activated by chemical compounds, acts as a transcription factor for the activation of drug-metabolizing genes and transporter genes. However, intensive studies have recently demonstrated its pleiotropic effects on cellular processes, including gluconeogenesis and hepatocyte proliferation. It is likely that the processes, such as drug metabolism, gluconeogenesis, and hepatocyte proliferation, triggered by CAR activation, are interrelated. Thus, liver hyperplasia in response to xenobiotic exposure may be part of the adaptation process: an increase in the liver is caused by the need for more detoxification enzymes. Proliferating hepatocytes require a building material; therefore, CAR-mediated reduction of gluconeogenesis can lead to the redistribution of glucose-6-phosphate into the pentose phosphate pathway. The pentose phosphate pathway provides hepatocytes with ribose for the synthesis of nucleotides, which are precursors for the biosynthesis of nucleic acids. At the same time, CAR-mediated suppression of gluconeogenesis may contribute to the maintenance of the required level of NADPH, which is also a product of the pentose phosphate pathway, to perform the protective function of drug metabolism under conditions of limited energy consumption. Nevertheless, several researchers have suggested CAR as a therapeutic target for glucose level correction or improving liver regeneration. This necessitates a better understanding of the signaling mechanisms that are triggered when CAR is activated, because these can have a profound effect on numerous processes, from drug–drug interactions and toxicity responses to tumor promotion. Studies conducted in recent years have shown that many of the effects of CAR are mediated through noncanonical signaling. In this regard, knowledge of such “nontraditional” mechanisms of gene regulation with the participation of CAR is very important. In our opinion, subsequent discoveries over the next few years will be made outside the “canonical” mechanism of CAR-mediated gene regulation.
